# Sometimes It’s Personal: Differential Outcomes of Person vs. Job at Risk Threats to Job Security

**DOI:** 10.3390/ijerph18147379

**Published:** 2021-07-10

**Authors:** Nicole Carusone, Rebecca Pittman, Mindy Shoss

**Affiliations:** 1 Department of Psychology, University of Central Florida, Orlando, FL 32816, USA; pittmanr03@knights.ucf.edu (R.P.); mindy.shoss@ucf.edu (M.S.); 2 Peter Faber Business School, Australian Catholic University, Melbourne 3065, Australia

**Keywords:** job insecurity, identity threat, self, organization based self-esteem

## Abstract

The current paper expands an under-addressed concept within the job insecurity literature, namely, whether threats to job security are specific to the jobholder (person-at-risk threats) or specific to the job (job-at-risk threats). Using a between-person experimental vignette design, 136 employed participants were asked to imagine themselves in either a Person-at-Risk or a Job-at-Risk scenario. As expected, participants in a person-at-risk scenario indicated more negative reactions to job insecurity, as captured by greater anticipated negative affect and poorer perceived social exchanges and organization-based self-esteem. They also reported reduced intention for interpersonal citizenship behavior and greater intention to engage in one form of impression management compared to individuals in a job-at-risk scenario. We interpret these findings in terms of their implications on individual versus group identity, as well as on well-being and the behavioral consequences of job insecurity.

## 1. Introduction

Job insecurity reflects a perceived threat to the continuity and stability of one’s employment as it is currently experienced [1,2]. Research amassed over the past few decades has revealed a wide range of negative consequences associated with experiencing job insecurity, such as poor psychological well-being and somatic health [3]. Job insecure employees also report poorer social exchange relationships with their organizations, poorer job attitudes and greater intention to quit [4,5].

However, these findings are far from uniform, perhaps due to the breadth of the job insecurity construct [2]. For example, researchers have distinguished between concern regarding the job as a whole, and concern regarding job features [1,6]. Both types of job insecurity have been found to lead to poor well-being, although some research suggests that the negative effects of quantitative (job loss) job insecurity may reach farther than qualitative (job feature loss), impacting home as well as work life [7]. A lesser-discussed but potentially important distinction concerns the perceived foci of the threat. Recently, Shoss built on Jacobsen and Hartley’s discussion of job threats to suggest two foci of job insecurity threats: the person and the position [2,8]. Specifically, individuals may perceive a job to be threatened regardless of the holder of the job (labeled job-at-risk, JAR), such as when entire departments are being laid-off. Alternatively, the perceived experience of job insecurity may be dependent on the person, meaning that the individual’s employment may be threatened although the position itself will be preserved (labeled person-at-risk, PAR). For example, individuals may feel they are at risk of being fired for inadequate performance or because of a poor relationship with their boss. Such PAR threats may be particularly pronounced in the United States, where much employment can be characterized as “at-will”. In contrast, mass layoffs due to business closures would reflect JAR threats. 

Although the person-at-risk versus job-at-risk insecurity concept has been briefly discussed in the literature, it has not yet been investigated in empirical work, and is deserving of greater theoretical and empirical attention. The goal of this paper is to offer new insights into the idea that there are different types of job insecurity threats—i.e., threats specific to the person and threats specific to the job—by examining their differential impacts on outcomes. In particular, we argue that the difference between these two perceived foci of job insecurity can be fairly pronounced and are likely very different experiences for individuals. Leveraging research on the self [9,10,11,12], we argue that JAR and PAR threats can have differing effects on job insecurity outcomes. We present an experimental vignette study conducted in the United States to examine how JAR and PAR threats may differentially relate to outcomes. This research points to the need for job insecurity research to examine the perceived foci of threats, and provides insight into threat foci as one potential factor underlying the variability in job insecurity results. This research is also of practical relevance for understanding how people perceive threats to job security.

## 2. Person-at-Risk (PAR) and Job-at-Risk (JAR) Threats to Job Security

Perceptions of job insecurity may be triggered by a variety of factors. Insecure employees may be responding to global events such as unemployment rates, changes within one’s organization, personal circumstances or group membership, among many other potential factors [2,13,14]. Although it is understood that job insecurity is a subjective experience that is not triggered by the same factors for every person, little research has sought to understand the different foci of threats tied to these circumstances. 

Reflecting on the widespread economic and organizational restructuring occurring across many countries in the late 1980s, Hartley, Jacobson, and colleagues conducted several studies to shed light on the job insecurity construct, thereby laying the foundation for future research on job insecurity [15]. In this volume, Jacobson and Hartley commented on the heterogeneity of job insecurity threats by noting (p. 9):

*“Threat to job security in the case of these employees [permanent employees] are of two types: a threat to the loss of the job regardless of the job-holder—through, for example, retrenchment, mergers, restructuring or the introduction of advanced technologies. Alternatively, job insecurity may arise through the loss or erosion of employment rights, for example, through changed contracts of employment. In the latter case, the job continues but the job-holder is vulnerable.”*

Their exploratory survey results found evidence of this distinction. Although many employees pointed to macrolevel factors such as demand for products as a source of insecurity, some also pointed to individual factors that may put them at risk, including age, work effort, and relationship with colleagues/supervisors. Thus, while some employees focused on the risk to the job independent of the job holder, others saw themselves as job holders to be at risk.

Shoss called for the field to examine the impact of such job threat foci on responses to job insecurity [2]. She labeled threats to the person as PAR (person-at-risk) threats, emphasizing that the threat is to the particular job holder. Alternatively, in JAR (job-at-risk) threats, the threat is to the position independent of the person [2,8]. In other words, under PAR threats, the job position itself remains, although the individual’s employment is terminated. For instance, an employee may be fired because they are performing below expectations or because of a personal conflict with their manager. In these situations, the individual will lose their job, but the position itself will remain and that individual will be replaced by a new employee. In JAR threats, it is the job position itself that disappears regardless of the job holder. This may be a result of a company-wide downsizing, where an entire team or department is being eliminated. A position may also be outsourced or automated, leading to the removal of the job altogether. Under a JAR threat, the job itself is being threatened, regardless of the person who currently holds it. The employee will still lose their job, but no one will be hired to replace them. While the outcome of both threats is the same (i.e., job loss), the surrounding circumstances are quite distinct.

The call for research on the differential outcomes of JAR and PAR threats is well-timed, given that emerging research in the job insecurity literature has found several “person-level” threats to job security, including people’s performance, quality of their relationships with others, and vulnerable status (e.g., minority, older, pregnant) [14,16,17,18]. Moreover, although a great deal of research has found negative outcomes of job insecurity in the workplace, including lowered performance and increased counter-productive work behaviors [19,20,21,22,23], these findings are not necessarily consistent across the literature. For example, others have found that job insecurity was associated with lower levels of withdrawal [24], greater performance [25,26], and lower counterproductive work behavior [27]. Others have found no relationship between job insecurity and work outcomes [28,29,30,31]. Although most research points to job insecurity as being detrimental for employee well-being and job attitudes, meta-analyses also indicate considerable variability in the strength of these relationships [5]. We posit that some of these differences may be caused by differences in the circumstance or foci of the threat itself.

## 3. Differential Impacts of PAR and JAR Job Insecurity

We argue that in comparison to JAR threats, perceptions of PAR threats are deeply personal, because they concern the specific employee’s value to the organization. Research on identity suggests that individuals hold multiple identities that are different from one another in various ways [32], including the degree to which each of these identities is important, or salient, to the individual [33]. Social psychology research suggests that an individual’s identity consists of three selves, the individual self, the relational self, and the collective self [9,10,11,12]. The individual self reflects an individual’s uniqueness on a variety of aspects, such as personality, goals and experiences, and distinguishes one individual from another. The relational self reflects an individual’s interpersonal relationships with those close to them and the roles of the individual within those relationships. The collective self refers to an individual’s membership in core social groups such as an organization [34]. Note that the self-concept represents an individual’s perceptions of themself based formed through experiences [35]. Self-esteem, in contrast, reflects a judgement of the goodness or badness of the self-concept. 

The framework of multiple selves provides a useful way of thinking about job insecurity experiences and, importantly, can be leveraged to suggest that people’s reactions to job insecurity may differ depending on whether they are faced with a person-at-risk or job-at-risk threat. With the singular employee being threatened, PAR threats can call into question personal characteristics such as one’s ability to do their job well or their value and worth as an employee. For example, if an accountant is being laid off because of poor performance, their individual self-concept as a capable worker is being threatened. JAR threats, however, are more likely to threaten one’s collective self as these threats are more generalized to the job itself regardless of the individual employed in that job. If instead the accountant is being laid off because their organization is outsourcing the accounting department, their collective self-concept as a member of the accounting department is being threatened rather than their own personal identity. Thus, because of the personal nature of PAR threats, these situations can be thought of as threats to the person’s ego and their personal identity. In contrast, JAR scenarios serve as threats to a person’s collective or social identity [34]. 

Petriglieri defines identity threat as “experiences appraised as indicating potential harm to the value, meanings, or enactment of an identity,” [32] (p. 644). In the case of job insecurity, threats that an individual appraises as threatening to their personal identity threaten an aspect of the employee’s identity that is much more salient to them than threats appraised as targeting the job. Therefore these threats are much more likely to cause significant harm. According to the content process of Nehrlich et al. [36], people prefer the individual self over the relational and collective selves, because it is more agentic in content, i.e., it sets them apart from others to express their uniqueness. Indeed, in a series of four studies utilizing various methods and cultures, Gaertner et al. [34] found the emergence of a three-tier motivational hierarchy of the selves. The individual self was at the top of the hierarchy, followed by the relational self and lastly, the collective self. In other words, individuals (a) experienced stronger reactions to, (b) avoided threats to, (c) donated more money towards the maintenance of, and (d) associated future goals with their individual self over the collective and relational self (see also [36]). 

We expect that these differences have important implications for how employees respond to JAR and PAR threats. In particular, because PAR threats are more central to the individual self, we anticipate that individuals will have a stronger reaction to these threats, compared with JAR threats, and engage in more action to try to protect themselves against these threats. 

## 4. Current Study

The current study aimed to investigate the differential effects of PAR and JAR threats to job security across a variety of personal and job-related outcomes. This study utilized a between-subjects experimental vignette study. Vignette studies allow for the manipulation of variables of interest [37], thereby providing insights into causal relationships [38]. As the goal of the study was to carefully isolate the effects of PAR as compared to JAR threats to job security, the study employed a paper people vignette study as recommended by Aguinis and Bradley [38]. This methodology involves asking participants to read a vignette and then indicate how they would respond on a variety of measures. As Aguinis and Bradley [38] note, a paper people vignette methodology allows for explicit control over the manipulated independent variable (i.e., PAR vs. JAR threats), while in this case avoiding confounding or ambiguous circumstances that might otherwise be present when investigating real-life job insecurity threats. 

Through the vignette design, we examined three broad categories of outcomes [2]. First, we selected several outcomes associated with employee well-being, including negative affect, organization based self-esteem, and counterproductive work behavior to reflect the negative well-being consequences of job insecurity. The choice of these variables aligns with past research that has found that ego or person-related threats in the workplace harm well-being and self-esteem [39,40]. For example, Selenko et al. found that job insecurity was associated with weaker social identity, which in turn affected well-being [41]. Research also suggests that identity threats at work can lead to increased misbehavior at work, with the logic that negative reactions are expressed via misbehavior [29,42]. Additionally, experimental research in social psychology finds that being devalued is associated with anger and sadness [43]. 

Second, we included quality of social exchange relationships [44], organizational citizenship behaviors, and turnover intention to capture potential impacts on the employee-employer relationship. These are common variables in organizational research emanating from the social exchange perspective, including research on job insecurity [23,45].

Third, we included work effort and impression management as potential behaviors indicative of efforts to try to counter threats (i.e., job preservation behaviors), which may reflect efforts to ingratiate oneself with one’s supervisor, demonstrate one’s worth to the organization, or compete with others. Although we anticipated that people responding to PAR threats would have more negative reactions in terms of well-being and social exchanges than people responding to JAR threats, we would also anticipate that they would be more motivated to engage in behaviors indicative of job preservation. This prediction follows from the finding of Gaerter et al., i.e., that people are more motivated to counteract threats to the personal than collective identity. Based on the logic described above, we hypothesized:

**Hypothesis** **1.** *PAR threats will be associated with poorer well-being compared with JAR threats, as indicated by (a) greater negative affect, (b) lower organization-based self-esteem, (c) greater instances of CWB-O, and (d) greater instances of CWB-I.*

**Hypothesis** **2.** *PAR threats will be associated with poorer social exchange relationships compared with JAR threats, as indicated by (a) poorer social exchanges with supervisors, (b) poorer social exchanges with one’s organization, (c) greater turnover intention, (d) fewer instances of OCB-O, and (e) fewer instances of OBC-I.*

**Hypothesis** **3.** *PAR threats will be associated with greater job preservation efforts compared with JAR threats, as indicated by (a) greater work effort and (b) greater impression management.*

**Figure 1:**
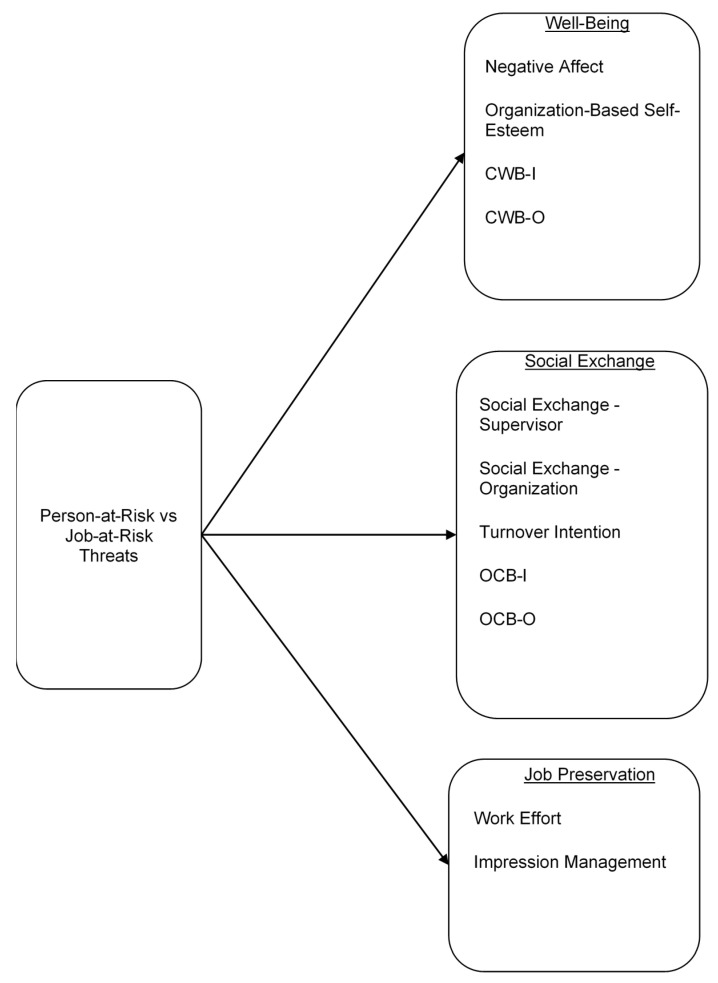
Conceptual model of person-at-risk and job-at-risk threats.

A conceptual model of our hypotheses can be seen in Figure 1.

## 5. Materials and Methods

### 5.1. Method

This study utilized a between-subjects experimental vignette study with a sample of employed individuals who were also enrolled in classes in a large southeastern university in the United States, conducted in Fall 2019. Participants were recruited via a university-wide research recruitment system (SONA) and completed the study online through a web-based survey platform (Qualtrics). Participants were randomly assigned via automated randomization to read one vignette depicting either a job-at-risk or person-at-risk job insecurity threat scenario. After reading the vignette, participants were asked to respond to a manipulation question that asked them to indicate whether the threat in the scenario they read was due to factors related to the employee (PAR) or factors related to the job (JAR). After responding to this manipulation check, participants were asked to complete survey measures imagining they were the employee in the vignette.

### 5.2. Vignettes

In line with the recommendations of Monin and Oppenheimer for stimulus sampling in vignette studies [46], the research team originally developed eight vignettes based on a content analysis from free response survey items from a pilot qualitative study on sources of job insecurity [47]. In this study of qualitative responses, participants were asked to indicate the sources of their job insecurity in an open-ended question. The most common responses highlighting PAR and JAR factors were used to create the narratives for eight initial vignettes. Four of the vignettes demonstrated job insecurity due to the most commonly reported factors related to the jobholder (i.e., poor job performance, inability to meet changing job demands, an aging employee, and challenging the status quo) in the pilot data (e.g., one pilot participant’s response was “yeah my boss caught me sleeping several times,” which is integrated into the poor job performance vignette). The remaining four vignettes provided examples of job insecurity due to the most commonly reported factors related to perceived threats to the job itself (e.g., economy, technology, organizational changes, and company illegal activities).

Based on an examination of scenarios across the conditions to identify if any of the scenarios were more difficult to identify as PAR or JAR, two scenarios were removed, one PAR scenario involving changing job demands and one JAR scenario involving job insecurity due to company illegal activities. These explanations had been noted in our pilot study, but with less frequency than the other PAR or JAR situations. The six vignettes retained for analysis can be seen in Appendix A.

### 5.3. Participants

In this study, 185 participants responded to questions about the six vignettes, of which 49 were removed for failing to respond correctly to the manipulation check. The final sample size was therefore 136 participants. The mean age of the final sample was 21.44 (*SD* = 5.82). They worked an average of 22.65 h per week (*SD* = 11.18) and had a mean work experience of 3.96 years (*SD* = 4.95). There were 83 females in the final sample (61.5%). Participants held a wide range of job titles such as chief operations officer, assistant manager, bartender, data input specialist, and attorney.

In order to check the equivalency of participant demographics across the randomized conditions, independent samples t-tests were conducted on participants’ assigned condition and the demographic variables. There were no significant differences between those assigned to the PAR condition and those assigned to the JAR condition in age, (*t*(133) = −0.825, *p* = 0.411), hours worked per week (*t*(134) = −1.123, *p* = 0.264), or work experience (*t*(134) = −0.762, *p* = 0.447). This supported the equivalency of the randomization.

### 5.4. Outcome Measures

Negative Affect. Anticipated negative affect was measured using the 10-item Negative Affect scale from PANAS, as proposed by Watson, Clark, and Tellegen [48]. Participants were asked to respond with how they would feel in response to the scenario they read and indicate the extent to which they believed they would experience a series of negative feelings and emotions on a 1 (very slightly or not at all) to 5 (extremely). Examples of the emotions are distressed, hostile, and nervous (α = 0.904).

Organization Based Self-Esteem. Anticipated organization based self-esteem was measured using the 10-item Organization-Based Self-Esteem measure proposed by Pierce, Gardner, and Cummings [49]. This scale measures the extent to which employees receive positive messages from the actions and behaviors from their managers and supervisors, such as “I count around here” and, “I am important.” Participants were asked to respond with the extent to which they would feel these messages on a 1 (strongly disagree) to 5 (strongly disagree) scale (α = 0.959).

Counterproductive Work Behavior. Anticipated interpersonal and organization directed counterproductive work behavior (CWB-I, CWB-O) was measured using the Interpersonal and Organizational Deviance Scale, as proposed by Bennett and Robinson [50]. The scale includes seven items assessing deviant work behaviors targeting individuals (e.g., “Make fun of someone at work”; α = 0.957) and twelve items assessing deviant work behavior targeting the organization (e.g., “Take property from work without permission”; α = 0.940). Participants were asked to respond with the extent to which they would engage in each of the behaviors on a 1 (Never) to 5 (Very Often) scale.

Social Exchange Relationship. Anticipated social exchange was measured using the Social Exchange Relationship Scale, as defined by Colquitt et al. [44]. Items measure how an employee characterizes their work relationship with their supervisor and organization based on mutual obligation, mutual trust, mutual commitment, and mutual significance on a 1 (strongly disagree) to 5 (strongly agree) scale. Cronbach’s alpha for the supervisor subscale was 0.913. Alpha for the organization subscale was 0.897.

Turnover Intention. Anticipated turnover intention was measured using a single item from Spector and Jex [51]. Participants were asked to what extent they would think about quitting their job on a 1 (not at all) to 5 (very much) scale.

Organizational Citizenship Behavior. Anticipated interpersonal and organization directed organizational citizenship behavior was measured using the OCB-I and OCB-O scales, as proposed by William and Anderson [52]. We used six items from the OCB-I scale (e.g., “Help others who have been absent”; α = 0.887) and seven items from the OCB-O scale (e.g., “Adhere to informal rules devised to maintain order”; α = 0.817). Participants were asked the extent to which they would engage in each of the behaviors on a 1 (never) to 5 (very often) scale.

Work Effort. Anticipated work effort was measured using a five-item scale adapted from de Jong and Elfring to capture individual work effort [53]. Participants were asked to what extent they would engage in each of the behaviors in the next three months on a 1 (completely disagree) to 5 (completely agree) scale. An example item was, “I would carry my fair share of the overall workload.” Cronbach’s alpha for this sample was 0.949.

Impression Management. Anticipated impression management was measured using an impression management scale proposed by Bolino and Turnley [54]. The scale contains five subfactors, self-promotion (e.g., “Talk proudly about your experience or education”; α = 0.907), ingratiation (e.g., “Complement your colleagues so they will see you as likeable”; α = 0.904), exemplification (e.g., “Try to appear like a hard-working, dedicated employee”; α = 0.839), intimidation (e.g., Let others know you can make things difficult for them if they push you too far”; α = 0.937), and supplication (e.g., “Try to gain assistance or sympathy from people by appearing needy in some area”; α = 0.941). Each subfactor had five items. Participants were asked how often they would engage in each of the behaviors over the next three months on a 1 (never behave this way) to 5 (often behave this way) scale.

### 5.5. Analytic Strategy

In order to test the hypotheses, multivariate analyses of variance (MANOVAs) and post hoc tests with Bonferroni correction were conducted to test the differences in outcome variables between participants presented with the PAR threat and JAR threat vignettes using the SPSS software program. Pairwise deletion was used to exclude participants with missing data on an analysis by analysis basis. Three MANOVAs were conducted, with the outcomes being grouped under the three categories of our conceptual framework: anticipated well-being, anticipated social exchange, and anticipated job preservation. In supplementary analyses, the dataset was split based on the assigned condition and one-way ANOVAs were conducted on the three different vignette scenarios within each condition in order to examine whether there were any differences in our outcome variables based on the specific scenario presented to the participants.

## 6. Results

### 6.1. Comparisons between PAR and JAR Conditions

Table 1 shows the results of the hypothesis testing. In support of Hypotheses 1, individuals in the PAR condition reported worse anticipated well-being compared with those in the JAR condition, *F*(4, 129) = 6.97, *p* < 0.001, Wilks’ Lambda = 0.82. Specifically, participants in the PAR condition reported higher negative affect (*F*(1,132) = 12.83, *p* < 0.001) and lower organization based self-esteem (*F*(1,132) = 21.67, *p* < 0.001) than those in the JAR condition. Participants in the PAR condition also reported that they would engage in more CWB-O behaviors (*F*(1, 132) = 2.49, *p* = 0.021) and CWB-I behaviors (*F*(1, 132) = 1.69, *p* = 0.047) than those in the JAR condition.

Hypothesis 2 was partially supported. Overall, individuals presented with a PAR scenario reported significantly poorer anticipated social exchange relationships (*F*(5, 128) = 5.71, *p* < 0.001, Wilks’ Lambda = 0.82). Specifically, in support of Hypothesis 2 a and b, participants in the PAR condition anticipated significantly poorer social exchanges with their supervisor (*F*(1, 132) = 24.63, *p* < 0.001) and the organization (*F*(1,132) = 11.89, *p* = 0.001). Hypothesis 2d was also supported, with participants in the PAR condition reporting lower OCB-I (*F*(1, 132) = 9.53, *p* = 0.002) than participants in the JAR condition. However, Hypotheses 2 c and e were not supported, as there were no significant differences in turnover intention or OCB-O between the two groups.

Hypothesis 3 was also partially supported. Overall, participants reported greater anticipated job preservation under the PAR condition compared with the JAR condition, *F*(6, 128) = 3.49, *p* = 0.003, Wilks’ Lambda = 0.86. In partial support of Hypothesis 3b, individuals in the PAR condition reported higher supplication impression management than participants in the JAR condition (*F*(1, 133) = 8.36, *p* = 0.004). However, there were no significant differences in the other impression management subscales (promotion, ingratiation, exemplification, and intimidation) work effort (Hypothesis 3a).

### 6.2. Supplemental Analyses

Supplemental one-way ANOVAs revealed significant differences between the PAR vignettes for IM Exemplification (*F*(2,52) = 4.53, *p* = 0.015). Results of Bonferroni’s post hoc test showed that individuals in presented with a vignette about an aging employee (*M* = 3.91, *SD* = 0.60) responded differently than participants presented with a vignette about poor job performance in terms of IM exemplification (*M* = 3.04, *SD* = 1.01; *p* = 0.012). Those presented with a vignette about challenging the status quo (*M* = 3.29, *SD* = 0.84) did not respond differently than those presented with either a scenario about an aging employee or a scenario about poor job performance. There were no significant differences between the PAR vignettes for any of the other outcome variables.

Differences between the three JAR vignettes were also examined. A series of one-way ANOVAs showed there were no significant differences between the three vignettes on any of the outcome variables.

## 7. Discussion

This study aimed to examine potential differential responses to PAR and JAR threats to job security. Our findings revealed several notable differences in line with our anticipation that PAR job insecurity serves as a more severe threat and thus would be associated with amplified responses. Individuals faced with the PAR scenarios reported poorer anticipated well-being, including greater negative affect, lower organization-based self-esteem, and greater intention to engage in organization-directed and interpersonal-directed counterproductive work behaviors than individuals faced with a JAR scenario. Individuals in the PAR conditions also reported poorer social exchange outcomes compared with the JAR condition, anticipating poorer relationships with their supervisor and the organization, as well as fewer interpersonal-directed organizational citizenship behaviors. Finally, under PAR threats, participants reported higher levels of some potential job preservation strategies, anticipating they would engage in more supplication impression management than those facing JAR threats.

These findings are consistent with our arguments that PAR threats may be more central to people’s identities than JAR threats and therefore elicit stronger outcomes. Although it is likely that both PAR and JAR threats are viewed negatively (as may be suggested by the means of our outcome variables across the two conditions), PAR threats may be more likely to induce feelings of lower self-worth given the threat to the person as a unique individual as opposed to the less salient collective or relational self as a member of that organization [34]. PAR threats may be taken by an individual to mean that they, as unique individuals, are inadequate to belong in the organization. On the contrary, JAR insecurity that threatens an individual’s place in an organization targets factors besides an individual’s uniqueness and instead are results of external factors such as the economy.

Additionally, employees in the PAR condition also reported that they would engage in more CWB-O behaviors than employees in the JAR condition. This aligns with previous findings that various types of identity threat can lead to CWB-O. For example, in three samples of employees from a transportation company, a public school system, and a municipality, identity threat was associated with antisocial behavior, revenge attitudes, hierarchical status and aggressive modeling [42,55]. Thus, in line with findings that PAR threats have greater negative impacts on well-being and social exchanges, individuals exposed to PAR threats may be more likely to act out against their organization.

Surprisingly, OCB-I was higher for individuals in the JAR condition, while there were no significant differences in OCB-O. The findings for OCB-I might indicate greater competition under PAR condition (i.e., not wanting to help people because there is a feeling that others are competing for a job), in line with previous studies in the job insecurity literature [2]. Alternatively, higher OCB-I could be interpreted as greater collective coping under JAR conditions. Callea et al. found that the relationship between job insecurity and OCB was fully mediated by organizational identification [56]. In a vignette study it may be difficult to induce conditions of organizational identification, which may be influenced by job tenure or other organizational characteristics that impact the development of identification [57].

Contrary to the hypotheses, most of the impression management strategies showed no significant differences between PAR and JAR conditions. The only significant difference was in supplication, whereby individuals show their weaknesses or their vulnerability [58]. The reported intended use of supplication may be a tactic to elicit empathy from organizational members. Alternatively, it could be an individual’s acceptance of their shortcomings. Research on occupational stigma, a different form of identity threat, suggests that acceptance of an individual’s stigmatized status may be one method by which individuals cope with their stigmatized identity [59]. Similarly, individuals experiencing PAR threats may accept their shortcomings as a way of maintaining the status quo [60].

### 7.1. Practical Implications

Our findings have the important practical implication that the target of the job insecurity (the person or the job position) may be important for shaping outcomes. In this sense, they also have implications for managers and organizations looking to understand and perhaps mitigate negative effects of job insecurity amongst their employees. While it may seem intuitive for organizations to target employees’ job insecurity during times of widespread change, such as a company merger, large reorganization, or downsizing, our findings suggest that it may be just as, if not more, important to focus on job insecurity during times of threats of individual termination or layoff. These individual PAR circumstances can be even more impactful to employees and lead to greater instances of negative behaviors compared to JAR threats. Thus, organizations would do well to monitor potential perceptions of PAR threats and to avoid instances where PAR threats may be induced (i.e., PAR threats could be induced through forced ranking systems or the routine use of firing to address poor selection decisions).

Based on these findings, organizations may benefit from interventions or policies that target individuals who may feel insecure about their jobs for reasons such as low performance reviews, altercations with management, increased workload, or other personal reasons. Such programs, such as an engagement survey or targeted training session, should aim to gauge these employees’ level of job insecurity and provide positive coping mechanisms or other information that can help mitigate negative outcomes during that time.

Additionally, organizations may utilize these findings to diagnose high levels of job insecurity among their workforce. In the absence of an obvious widespread threat, such as the aforementioned JAR scenarios, job insecurity may not be typically examined when trying to identify causes of pervasive performance issues or CWBs. However, in such cases it may be beneficial for organizations to examine if patterns of PAR threats causing high levels of job insecurity may be contributing to workplace well-being and morale issues.

### 7.2. Limitations and Future Research Directions

Future research should continue to explore differences in outcomes between PAR and JAR threats to job security. Although experimental vignette studies have many strengths, field research should examine whether PAR and JAR threats differentially impact behavior. Future research may seek to examine different conceptualizations of the outcomes, for example, using a measure of work stress instead of negative affect. The current study is also limited in that, although the vignettes allowed for relative comparison against PAR and JAR threats, there was no control condition to compare the effects of both threats against individuals without job insecurity. Therefore, we cannot fully know whether or not both PAR and JAR threats negatively impact the outcome variables. Future research should consider the extent to which PAR and JAR may impact a spectrum of job insecurity perceptions at both low and high levels of severity.

Another noteworthy limitation of our study is the degree to which participants had difficulty responding correctly to the manipulation check and identifying whether they were reading a PAR or JAR scenario. Interestingly, a higher percentage of incorrect responses occurred in the PAR scenarios compared with the JAR scenarios. This pattern poses the question of whether PAR threats are more difficult for individuals to perceive or acknowledge. As previously discussed, threats to the individual self can be quite psychologically damaging [32]. Since in a PAR situation, the individual is often the most culpable for the potential job loss, it is possible that individuals may have a difficult time acknowledging a PAR threat as such. While we chose to remove individuals who responded incorrectly to this item to avoid potential contamination, future research should examine potential differences in the way individuals perceive and acknowledge PAR versus JAR threats.

Our study is also limited in that we did not account for any mediating variables which may impact the relationship between the vignettes and outcome variables and help to explain effects. For example, future research might examine perceived threats to personal and collective identities. There might be other mediating mechanisms explaining specific effects as well. For example, one such variable that may explain the lack of findings regarding CWB-I is moral disengagement. Sahi and Ahmad found that moral disengagement mediated the relationship between job insecurity and both CWB-O and CWB-I but that the effects were stronger for CWB-O [61]. Individuals experiencing PAR threats may be more likely to blame the organization than to blame other individuals. Additionally, PAR threats may be viewed as posing a potential risk to people in the future. Individuals who perceive PAR insecurity may perceive future threats to themselves that extend beyond the longevity of their tenure in the job. That is, if an individual loses their job due to factors related to themselves, they may be concerned that they would not be successful in future jobs due to those same person-at-risk factors. Future research should examine variables which may mediate the relationship between PAR or JAR threats and various outcomes.

It would also be interesting to examine whether some PAR threats are seen as potentially more malleable, as well as if there are different ways that people try to counteract PAR and JAR threats. To speculate, if individuals seek to try to counteract PAR threats, they might try to make a convincing argument about themselves as suitable for the job and organization. If individuals seek to try to counteract JAR threats, they might try to make an argument about the necessity of the job for achieving the organizations’ goals (e.g., why a restaurant should still employ barkeepers when they have a barkeeping robot).

Another important consideration in future research on identity at work is how identity and identification is changing due to the COVID-19 pandemic. Ashforth argues that COVID-19 is changing the way in which individuals meet their need for identity due to more remote work and fewer face-to-face interactions [62]. He argues that individuals will define themselves less in terms of the organizational identity, instead relying on other forms of collective and individual identities. In the case of PAR and JAR job insecurity, this may mean that an individual’s identification with their organization over time may become less salient, while other identities such as personal identity become more salient. This increased reliance on individual identity over organizational identity could lead to more negative reactions from employees when that individual identity is threatened in cases of PAR job insecurity. Future research should continue to examine changes in the way people construct identities as well as methods individuals use to buffer the negative impacts of PAR threats.

## 8. Conclusions

This paper expands upon job insecurity research to elaborate on and investigate the differential outcomes of two foci of threats to job security: person-at-risk and job-at-risk. Through an experimental vignette design, this study found that person-at-risk threats to job security were associated with higher anticipated negative affect, i.e., lower organization-based self-esteem, greater intention to engage in counterproductive work behaviors, poorer quality of social exchanges with supervisors and the organization, and lower interpersonal citizenship behaviors, as compared with job-at-risk threats. These differing effects suggest that people may respond differently to person-at-risk or job-at-risk threats. We encourage future work to further explore the mechanisms of these foci of job insecurity, as well as their differential outcomes and boundary conditions.

## Figures and Tables

**Table 1:** Results of MAOVAs.

	Type of Job Insecurity Threat									
	Person-at-Risk			Job-at-Risk						
	M	SD	n	M	SD	n	Wilks’ Lambda	F	df	Adjusted *p* *
**Anticipated Well-Being**							**0.82**	**6.97**	**4, 129**	**<0.001**
Negative Affect	2.98	0.91	55	2.46	0.91	81		12.83	1, 132	<0.001
Organization Based Self-Esteem	2.89	1.04	55	3.70	0.93	80		21.67	1, 132	<0.001
CWB-O ^1^	1.71	0.86	55	1.43	0.54	81		2.49	1, 132	0.021
CWB-I ^2^	1.48	0.81	54	1.29	0.62	81		1.69	1, 132	0.047
**Anticipated Social Exchange**							**0.82**	**5.71**	**5, 128**	**<0.001**
Social Exchange—Supervisor	2.93	1.08	54	3.69	0.69	81		24.63	1, 132	<0.001
Social Exchange—Organization	2.79	1.06	54	3.38	0.86	81		11.89	1, 132	0.001
Turnover intention	329	0.96	55	3.25	1.00	80		0.14	1, 132	0.708
OCB-I ^3^	3.27	0.91	55	3.72	0.77	81		9.53	1, 132	0.002
OCB-O ^4^	3.83	0.95	55	4.00	0.64	81		1.42	1, 132	0.235
**Anticipated Job Preservation**							**0.86**	**3.49**	**6, 128**	**0.003**
Work Effort	3.91	1.15	55	4.16	0.84	81		2.31	1, 133	0.131
IM Supplication	2.13	1.13	55	1.64	0.85	80		8.36	1, 133	0.004
IM Self Promotion	3.10	1.00	55	3.03	0.94	80		0.19	1, 133	0.661
IM Ingratiation	3.10	1.01	55	3.13	0.94	80		0.03	1, 133	0.858
IM Exemplification	3.33	0.93	55	3.02	0.91	80		3.8	1, 133	0.053
IM Intimidation	1.90	1.01	55	1.71	0.80	80		1.48	1, 133	0.226

* *p*-values are adjusted for multiple comparisons using Bonferroni correction; ^1^ Organizational counterproductive work behavior; ^2^ Interpersonal counterproductive work behavior; ^3^ Interpersonal organizational citizenship behavior; ^4^ Organizational citizenship behavior. The bold in the table shows a distinction between the overall results and post-hoc results.

## Data Availability

The data presented in this study are available on request from the corresponding author.

## Appendix A. Study Vignettes

### Appendix A.1. Person-at-Risk Vignettes

Imagine you are an employee in your late 40 s at a large corporate chain. You have been with the same company for several years. Your job can be very physically demanding. You often come home sore and have begun having chronic back pain. Part of you wonders how long you will be able to continue with this line of work. Additionally, your company has recently started to make a push to hire younger employees. They have tried to covertly push out older employees and hire younger ones, but the older employees are aware of the company’s motives. They are able to pay younger employees lower salaries to do the same job. Right now, you are still able to meet the physical job requirements but you are noticeably slower than you were when you started several years ago. You are worried that they will fire you so that they can hire someone younger for a lower cost.

Imagine you are an employee at a large corporate chain. You have been with the same company for several years. The job relies heavily on individual contributions of each employee. In the last two months, your boss has caught you falling asleep at work a few times. Your boss has also noticed you coming into work late at least once a week or not coming in at all a few times without giving them notice. You have had to redo several tasks multiple times because they were not done to the satisfaction of your boss and the company. Your boss and coworkers are often correcting mistakes that you make in your work. During last year’s performance evaluations, you were told that your performance needed to improve. You are worried that your boss may say that you are an unreliable employee and have you fired.

Imagine that you are an employee at a large corporate chain. You have been with the same corporation for several years. You have noticed that employees seem hesitant to do anything outside of their explicit job duties. Employees do not try to operate outside of the status quo. You had a lot of great ideas for new innovations and company directions. You have tried to pitch some of your ideas to your supervisor and their immediate supervisor a few times but your supervisor told you that it was not your job to have these kinds of ideas and their supervisor just seemed uninterested. Recently, a coworker told you that your boss does not like anyone who challenges the status quo and has made up excuses to have several employees fired in the past for suggesting changes. Now you are worried that your supervisor will try to drive you out of the company too.

### Appendix A.2. Job-at-Risk Vignettes

Imagine that you are an employee at a large corporate chain. You have been with the same corporation for several years. The economy is in the middle of another recession. Sales are at an all-time low and your company is struggling financially. The company tried several cost cutting measures. Initially they tried changing the suppliers of products they use and buying cheaper products. However, they were not able to save enough money to match the decrease in sales. Next, they tried cutting several full-time employees down to part-time. You were lucky enough to not have your hours cut at first. However, the company was still struggling to stay afloat and ended up laying off several full and part time employees. The economy is still struggling and sales for your company continue to decrease. Everyone is worried that there will be another round of layoffs or cuts to hours. Some people have even hinted that the company may have to close for good if they cannot make major cuts to costs or improve their sales

Imagine that you are an employee at a large corporate chain. You have been with the same corporation for several years. You represent several authors in advertising and selling their works for a commission. You have witnessed society’s transition from paper books to electronic books over the last few years. Recently, however, there has been another shift. More and more people are either acting as their own agents to have their books published electronically on websites such as Amazon, or they are publishing their books themselves on their personal blogs and websites. This technological shift has resulted in fewer and fewer new clients for your agency. A few of your long-term clients have also left your company to publish their works themselves. It seems likely that your company will be completely replaced by self-publication or self-representation.

Imagine that you are an employee at a large corporate chain. You have been with the same corporation for several years. The owner of the company has decided to retire and sell the company. There is a lot of uncertainty in what the new owner of the company will do. They have acquired several companies in the past and have a track record for making major internal changes and outsourcing work to other countries. Some of your coworkers are worried that different departments will be collapsed or eliminated altogether while others are merged with other companies the owner has. There has already been a complete overturn in upper level management positions and the new managers are auditing all of the departments and individual employee contributions. You are particularly worried that your job may be moved overseas and do not know if you will be able or allowed to go with the job.
